# The Unpaved Road of Non-Coding RNA Structure–Function Relationships: Current Knowledge, Available Methodologies, and Future Trends

**DOI:** 10.3390/ncrna11020020

**Published:** 2025-03-02

**Authors:** Ana Lúcia Leitão, Francisco J. Enguita

**Affiliations:** 1 Departamento de Química, Faculdade de Ciências e Tecologia, Universidade NOVA de Lisboa, Campus da Caparica, 2829-516 Caparica, Portugal; aldl@fct.unl.pt; 2 Faculdade de Medicina, Universidade de Lisboa, Av. Prof. Egas Moniz, 1649-028 Lisboa, Portugal

**Keywords:** non-coding RNA, X-ray crystallography, cryo-EM, chemical probing, structure–function relationships

## Abstract

The genomes from complex eukaryotes are enriched in non-coding genes whose transcription products (non-coding RNAs) are involved in the regulation of genomic output at different levels. Non-coding RNA action is predominantly driven by sequence and structural motifs that interact with specific functional partners. Despite the exponential growth in primary RNA sequence data facilitated by next-generation sequencing studies, the availability of tridimensional RNA data is comparatively more limited. The subjacent reasons for this relative lack of information regarding RNA structure are related to the specific chemical nature of RNA molecules and the limitations of the currently available methods for structural characterization of biomolecules. In this review, we describe and analyze the different structural motifs involved in non-coding RNA function and the wet-lab and computational methods used to characterize their structure–function relationships, highlighting the current need for detailed structural studies to explore the molecular determinants of non-coding RNA function.

## 1. Prevalence of Non-Coding Genes in the Genomes of Complex Eukaryotes

The genomes of higher eukaryotic organisms contain information units that will be translated into polypeptides (coding DNA) and territories that will not encode for proteins (non-coding DNA). Non-coding DNA harbors a wide array of elements, such as regulatory regions (promoters, enhancers, silencers, and insulators), repetitive elements (transposons and satellite DNA), and structural regions (telomeres and centromeres), along with functional non-coding RNAs (nucleolar RNAs, ribosomal RNAs, transfer RNAs, microRNAs, PIWI-interacting RNAs, and long non-coding RNAs) [1]. The extensive prevalence of non-coding sequences in the human and complex eukaryotic genomes has profound evolutionary implications, reshaping our understanding of genetic complexity, regulatory innovation, and adaptive potential. One of the most striking evolutionary implications of abundant non-coding DNA is its contribution to regulatory complexity [2]. The human genome’s vast non-coding regulatory regions include enhancers, silencers, insulators, and ncRNA genes that orchestrate gene expression patterns, which are also supported by the pervasive transcription phenomenon [3]. These elements allow for the fine-tuned spatiotemporal regulation of protein-coding genes, which is also essential for the development and functionality of multicellular organisms [2]. The evolutionary plasticity of non-coding regions has likely facilitated the rapid emergence of adaptive traits. Unlike protein-coding genes, where mutations are often deleterious, mutations in non-coding regions are less constrained and more likely to be neutral or adaptive. This flexibility enables the exploration of novel regulatory networks and functions without disrupting essential protein-coding sequences [4]. The ncRNA-driven regulatory networks are hypothesized to drive much of the phenotypic complexity observed in higher eukaryotes. For instance, humans and other mammals have a similar number of protein-coding genes to simpler organisms, such as nematodes, yet they exhibit vastly greater biological complexity. This disparity could be partially explained by the regulatory information present within non-coding regions [5]. The human genome contains tens of thousands of non-coding RNA (ncRNA) genes, with long non-coding RNAs (lncRNAs) being the most represented family of genome information units. The last version of the human GENCODE database includes approximately 7500 small ncRNA and over 35,000 lncRNA genes [6].

NcRNA genes are transcribed to produce RNA molecules that will have different regulatory effects over the genomic output, controlling the flow of information from coding genes, and acting either at the nucleus or the cytoplasm [7]. As with any RNA molecule, ncRNAs simultaneously harbor sequence and structural information; however, their regulatory functions are largely dictated by their secondary and tertiary structural features rather than primary nucleotide sequences. The structural features of ncRNAs are responsible for their participation in molecular recognition phenomena, catalysis of enzymatic reactions, and the regulation of gene expression [8]. Characterizing the structure of ncRNAs by different techniques, either by wet-lab protocols or computer-based methods, is essential to dissect their functions and mechanisms of action.

## 2. General Principles and Rules Governing RNA Structure

**Figure 1:**
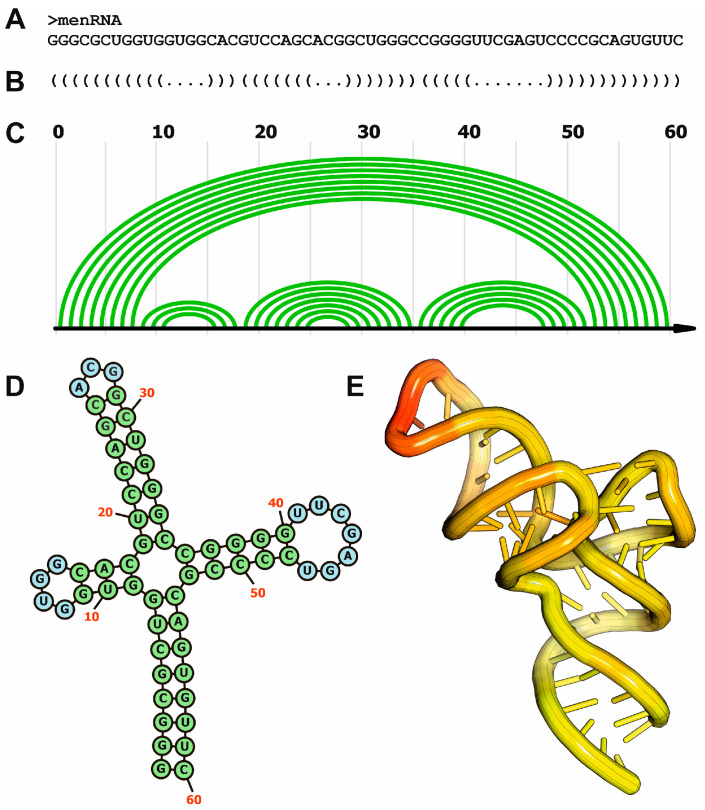
Different organization levels of RNA structure and their representations, exemplified by the human menRNA. (**A**) Primary structure of memRNA; (**B**) secondary structure representation of memRNA using the “dot-bracket” notation; (**C**) secondary structure of memRNA using the arc representation; (**D**) secondary structure of memRNA using the 2D diagram format; (**E**) tridimensional structure of memRNA using coordinate data extracted from the experimentally determined structure (PDB code: 8VT5).

The functional versatility of RNA relies on its ability to adopt diverse three-dimensional (3D) structures, mainly driven by the single-chain nature of RNA, and the presence of ribose sugars in the building nucleotide units. Understanding the underlying principles and features of RNA 3D architecture is specifically relevant for elucidating the biological roles of ncRNAs. Similar to proteins, RNA presents different organization levels, encompassing primary, secondary, and tertiary structures (Figure 1). The primary structure of RNA refers to its linear sequence of ribonucleotides and encodes the genetic and functional information of the RNA molecule. The RNA sequence determines the potential for base pairing and stacking interactions, which could drive the generation of higher-order structures [9]. Secondary structure arises from hydrogen bonding between complementary bases within the same RNA molecule, giving rise to several RNA motifs such as stems and loops [10,11]. In RNA molecules, due to the presence of a 5’-hydroxyl group in the ribose sugar, the base pairing phenomena can also be achieved by non-canonical rules (non-Watson-Crick pairing), expanding the repertoire of possible base interactions when compared to DNA [12]. Additionally, tertiary structure refers to the three-dimensional conformation of an RNA molecule, arising from long-range interactions between secondary structural elements. Key tertiary motifs include pseudoknots, formed by base pairing between a loop and a complementary sequence outside the loop; triple helices, or structures formed by interactions between three strands of RNA [13]; and kissing hairpins, structures that stabilize two hairpins by interactions between the unpaired looped bases [14,15].

The 3D structure of RNA is a result of intricate interactions among its nucleotide components. RNA molecules will fold into conformations that minimize their Gibbs free energy, achieving a stable structure under given environmental conditions [9]. The folding process reduces the conformational entropy of the molecule, being driven by favorable enthalpic interactions, including hydrogen bonding and base stacking. RNA folding typically occurs hierarchically, where secondary structures (e.g., helices and loops) form first, followed by tertiary interactions. The stability of RNA structures is maintained by a combination of noncovalent interactions that include base pairing, base stacking, hydrogen bonding, and electrostatic interactions [16]. Canonical base pairing provides significant thermodynamic stability to RNA structures. Hydrogen bonds between the bases in A-U (two hydrogen bonds) and G-C (three hydrogen bonds) pairs contribute to the overall stability of RNA duplexes and other base-paired regions. Canonical base pairing also plays a role in guiding the folding pathways of RNA. The complementarity of A-U and G-C pairs facilitates the correct alignment of RNA strands, minimizing misfolding and the formation of kinetically trapped structures [17]. This specificity is particularly critical in complex RNA molecules, such as ribosomal RNA, ribozymes, and lncRNAs, where precise folding is required for specific functions [18]. Base stacking arises from π–π interactions between the aromatic rings of adjacent nucleobases, complemented by hydrophobic forces that exclude water molecules from the stacked regions. These interactions are stronger in RNA than in DNA due to RNA’s 2′-hydroxyl group, which introduces additional electrostatic effects and influences sugar-phosphate backbone geometry [19]. Following the expanded nearest-neighbor model for the prediction of thermodynamic parameters, the base stacking phenomenon results in a substantial stabilization of RNA structures by lowering their free energy [20]. Unlike hydrogen bonds in base pairing, stacking interactions are largely sequence-independent but strongly related to the sequence context. Pyrimidine-purine sequences (e.g., C-G or U-G) tend to exhibit stronger stacking due to optimal overlap of aromatic π-electron clouds [21,22]. These interactions contribute to the enhanced stability of RNA helices, particularly in regions with high G-C content, as guanine and cytosine bases have favorable stacking geometries.

In addition to stabilizing Watson–Crick duplexes, base stacking also supports the stability of non-canonical structures, such as pseudoknots, internal loops, and junctions. These interactions provide a scaffold for tertiary interactions, such as metal ion coordination and long-range base pairings, that contribute to the overall folding of complex RNAs [23]. Base stacking also influences the conformational dynamics of RNA. Partially stacked regions enable the structural flexibility required for RNA to undergo conformational changes, which is critical for its interactions with proteins, small ligands, and other nucleic acids [24]. Moreover, hydrogen bonding extends beyond base pairing to mediate tertiary interactions that stabilize the three-dimensional architecture of RNA molecules. Hydrogen bonds between unpaired bases in RNA loops facilitate long-range interactions [14] and contribute to the stabilization of intricate tertiary folds when they are formed between bases and the sugar-phosphate backbone [25]. Additionally, the RNA backbone carries a negative charge at each phosphate group, which contributes to significant electrostatic repulsion within the molecule. This repulsion presents a thermodynamic challenge for the folding and stabilization of compact RNA structures [26,27]. The presence of cations in the cellular environment mitigates the repulsion between negatively charged groups. Counterions, such as sodium (Na^+^), potassium (K^+^), magnesium (Mg^2+^), and other cationic compounds such as polyamines, could neutralize the phosphate backbone charges, also contributing to stable RNA conformations [28,29].

## 3. Functional Relevance of Structural Elements in Non-Coding RNAs

**Figure 2:**
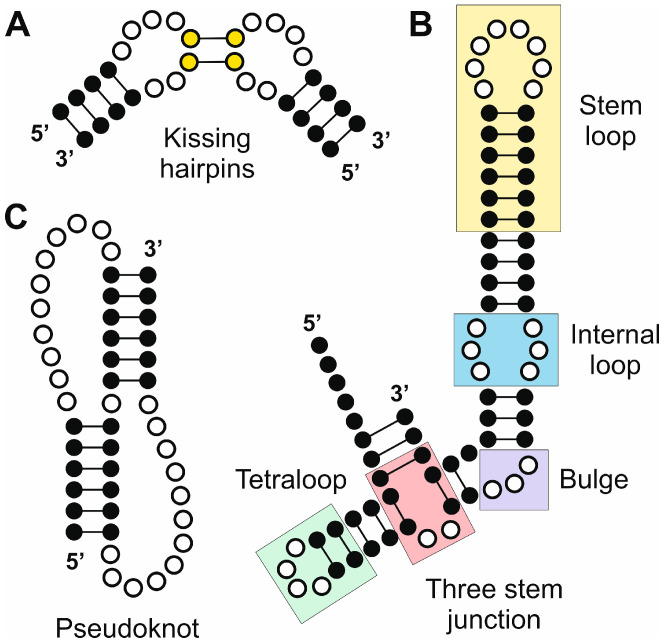
Selected RNA elements generated by secondary and tertiary structure arrangements with relevance in the biogenesis and function of ncRNAs. (***A***) Kissing hairpins; (**B**) combined RNA segment that contains a stem loop, and internal loop, a bulge, a three-stem junction and a tetraloop; and (**C**) pseudoknot.

RNA structural motifs are arrangements of nucleotide sequences and secondary structures that contribute to the overall architecture and function of RNA molecules. The structural arrangement of RNA motifs arises from specific nucleotide interactions. These motifs are characterized by recurrent patterns of base pairing, stacking, and backbone conformations. They can form within single-stranded regions or emerge from interactions between different secondary structure elements [30]. Structural motifs often stabilize RNA tertiary structures and facilitate interactions with proteins, small molecules, or other RNA molecules, being very relevant in the biogenesis and function of ncRNAs (Figure 2).

### 3.1. Hairpin Loops

Hairpin loops are the most common secondary structural motifs in RNA. They consist of a loop of unpaired nucleotides flanked by a double-stranded stem (Figure 2). The loop typically contains 4–10 nucleotides and stabilizes the RNA structure by closing the end of a stem. Often found at the terminus of a stem, hairpin loops are single-stranded RNA segments that loop back on themselves to form a stable structure [31]. The GNRA tetraloop, where G, N, R, and A represent guanine, any nucleotide, purine, and adenine, respectively, is one of the most common. This motif is related to the stabilization of RNA tertiary structures and can participate in long-range tertiary interactions, facilitating RNA folding and functionality [32].

Hairpin loops are central to the maturation process of microRNAs (miRNAs), a conserved class of small ncRNAs involved in post-transcriptional gene silencing. MiRNA precursors, known as primary miRNAs (pri-miRNAs), are transcribed by RNA polymerase II and fold into characteristic hairpin loop structures [33]. These structures are recognized and processed by the nuclear microprocessor complex, comprising the RNase III enzyme Drosha and its cofactor DGCR8. The hairpin loop ensures precise cleavage by the microprocessor, enabling the release of precursor miRNAs (pre-miRNAs) with defined stem-loop structures [34]. The microprocessor complex has structural flexibility to accommodate different pri-miRNAs [35]. Hairpin loops also regulate the efficiency and specificity of miRNA–target interactions. For instance, mutations in the hairpin structure of let-7 miRNA result in aberrant processing and diminished silencing activity [36]. These findings emphasize how the physical characteristics of hairpin loops contribute to the fidelity of miRNA maturation. Furthermore, auxiliary proteins such as the double-stranded RNA-binding protein DGCR8 and TAR RNA-binding protein (TRBP) recognize and stabilize hairpin loops, enhancing the precision of Drosha and Dicer cleavage events [37]. These protein–RNA interactions highlight the broader role of hairpin loops in facilitating multi-protein complexes essential for miRNA function.

Small interfering RNAs (siRNAs) also rely on hairpin structures for their generation and function. Endogenously derived siRNAs often originate from long double-stranded RNA (dsRNA) precursors, which can include hairpin-forming transcripts. Dicer processes these dsRNA precursors into siRNA duplexes [38]. The precise structure of the hairpin loop can influence Dicer activity, determining the efficiency and accuracy of siRNA generation [39].

Long non-coding RNAs (lncRNAs), which regulate diverse cellular processes such as chromatin remodeling and transcriptional control, also feature hairpin loops as functional motifs. Hairpin structures in lncRNAs can mediate protein–RNA interactions, RNA–RNA duplex formation, and scaffolding of ribonucleoprotein complexes [40]. For instance, the lncRNA HOTAIR utilizes hairpin loops to interact with polycomb repressive complex 2 (PRC2), guiding it to specific genomic loci for histone modification and transcriptional silencing [41]. Similarly, MALAT1, a nuclear retained lncRNA, contains hairpin structures that regulate its stability and interactions with splicing factors, highlighting the versatility of these motifs [42].

### 3.2. Bulges and Internal Loops

Bulges are regions of unpaired nucleotides on one strand of an RNA duplex, while internal loops consist of unpaired nucleotides on both strands (Figure 2). Bulges and internal loops are ubiquitous in ncRNAs, appearing in ribosomal RNAs (rRNAs), small nuclear RNAs (snRNAs), small nucleolar RNAs (snoRNAs), miRNA precursors, and lncRNAs. Both structural features can disrupt regular Watson–Crick base pairing, introducing flexibility and asymmetry into RNA structures [43]. This flexibility is critical for enabling ncRNAs to adopt specific conformations required for their functions. Bulges often act as hinge points, allowing the RNA to bend or twist into compact tertiary structures. This flexibility can be essential for the formation of higher-order assemblies, such as pseudoknots or long-range base-pairing interactions [8]. Internal loops, especially those with specific sequence motifs, can form non-canonical base pairs, such as G–U wobble pairs, or participate in hydrogen bonding networks that stabilize the RNA’s tertiary structure [15].

Bulges and internal loops are critical for RNA–protein interactions, which are fundamental to the functions of many ncRNAs. These structural elements often serve as recognition motifs for RNA-binding proteins (RBPs), which preferentially bind to unpaired or irregular regions of RNA [44]. For instance, bulges and internal loops within rRNAs are essential for their interactions with ribosomal proteins and other factors involved in translation [45]. The internal loops in the peptidyl transferase center of the ribosome mediate contacts with transfer RNAs (tRNAs) and catalytic proteins [46]. Additionally, internal loops in miRNA precursors (pre-miRNAs) are recognized by processing enzymes such as Dicer. A study by MacRae and Doudna described how Dicer endonuclease can establish specific interactions with these internal loops, highlighting their role in enzyme orientation for accurate substrate cleavage [47].

Bulges and internal loops in lncRNAs contribute to their ability to act as scaffolds for protein complexes. For example, the lncRNA Xist, which mediates X-chromosome inactivation, contains internal loops that facilitate interactions with the polycomb repressive complex 2 (PRC2). A detailed study by Zhao and coworkers highlights these interactions, demonstrating how specific structural motifs in Xist recruit PRC2 to silence the X chromosome [48]. Another interesting example involves the lncRNA MALAT1, which contains an internal loop that enhances its ability to interact with the splicing factor SRSF1, facilitating alternative splicing regulation [49].

### 3.3. Pseudoknots

Pseudoknots are a common and functionally significant structural motif found in RNA molecules. These structures occur when bases in a loop of single-stranded RNA form complementary base pairs with a sequence outside the loop, creating a complex tertiary architecture [50]. This interaction results in a compact, often stable three-dimensional structure. Despite their relative simplicity compared to larger RNA folds, pseudoknots play critical roles in the function of ncRNAs, influencing processes such as translation, catalysis, and genome stability, especially in complex organisms. Pseudoknots can be classified based on their topology and the number of stems and loops involved. The simplest form, known as the H-type pseudoknot, consists of two helical stems connected by loops [51]. More complex pseudoknots may involve additional stems, loops, or interactions with proteins and metal ions. The stability of pseudoknots is influenced by factors such as the length of the loops, the sequence composition, and the presence of stabilizing cations such as magnesium ions. The dynamic nature of RNA allows pseudoknots to fold and unfold, enabling them to serve as molecular switches in various biological processes [52].

In many viral and cellular coding RNAs, pseudoknots promote programmed ribosomal frameshifting, a mechanism that allows the ribosome to change reading frames during translation [53]. Pseudoknots positioned downstream of a slippery sequence in the mRNA create mechanical tension as the ribosome unwinds them, stalling the ribosome and inducing frameshifting [50]. This mechanism is critical for the regulation of gene expression in some RNA viruses and for the synthesis of viral polyproteins [54].

Pseudoknots can also act as scaffolds for assembling multi-molecular complexes. In snoRNAs, pseudoknots help organize and stabilize interactions with proteins and other RNAs involved in ribosomal RNA processing and modification. This scaffolding role is essential in complex organisms where ncRNAs participate in highly coordinated molecular machineries [50]. In lncRNAs, pseudoknots contribute to their ability to act as molecular decoys, scaffolds, or guides for proteins and other RNAs. A relevant example is the telomerase complex, which contains a 451 nt ncRNA (TERC RNA) together with the enzymatic core of the reverse transcriptase (hTERT). TERC ncRNA contains a series of conserved domains, including a pseudoknot. The TERC pseudoknot has been described as an essential feature for the stabilization of the telomerase complex and its processive activity [55]. The stability and dynamics of pseudoknots in specific lncRNAs are also involved in cellular responses to some signals, highlighting their importance in the regulation of gene expression networks in complex organisms. For example, in the MEG3 lncRNA, a human maternally expressed tumor suppressor that regulates the p53 pathway, an evolutionarily conserved region contains two distal motifs that interact by base complementarity to form alternative, mutually exclusive structures. Mutations that disrupt these interactions impair MEG3-dependent p53 stimulation in vivo and disrupt MEG3 folding in vitro [56].

### 3.4. Kissing Hairpins

Kissing hairpins refer to a specific tertiary interaction between two RNA hairpins, wherein the single-stranded loop regions of the hairpins interact by base pairing to form a stable duplex (Figure 2). This motif is stabilized by complementary sequences within the loops, leading to the formation of a “kissing” interaction. These structures are stabilized by canonical Watson–Crick base pairing and sometimes further reinforced by stacking interactions and cation coordination [30].

The formation of kissing hairpins contributes to the structural stability of lncRNAs in several ways. First, these interactions reduce the overall entropy of the molecule by locking dynamic regions into defined conformations [57]. This stabilization is particularly important for lncRNAs exposed to the intracellular milieu, where RNAses and other destabilizing factors are prevalent. For example, studies on the lncRNA MALAT1 (metastasis-associated lung adenocarcinoma transcript 1) suggested that such tertiary interactions protect the RNA against exonucleolytic degradation [58]. Additionally, kissing hairpins may influence lncRNA folding pathways, guiding the molecule into functionally active conformations. This folding precision ensures that lncRNAs can interact with their protein, DNA, or RNA targets effectively. For instance, a kissing hairpin motif in the HOTAIR (HOX transcript antisense intergenic RNA) lncRNA facilitates its scaffold function, enabling interactions with chromatin-modifying complexes [59].

Beyond structural stability, kissing hairpins play active roles in the functional repertoire of lncRNAs. These motifs serve as interaction platforms for RNA-binding proteins (RBPs) and other nucleic acids. Their defined geometry and sequence specificity make them ideal docking sites for RBPs, as observed in the interaction of the lncRNA Xist with its associated proteins [60]. Kissing hairpins also facilitate lncRNA-mediated RNA-RNA interactions. In the case of lncRNAs involved in RNA splicing, such as NEAT1, kissing hairpin motifs contribute to the assembly of paraspeckles by mediating interactions between NEAT1 and other RNAs [61]. The conservation of kissing hairpin motifs among different lncRNAs suggested an evolutionary trend that could have functional implications. Comparative analyses have also revealed conserved loop–loop interactions in lncRNAs across species, underscoring their functional importance [62].

## 4. Methods and Protocols to Study ncRNA Structures

**Figure 3:**
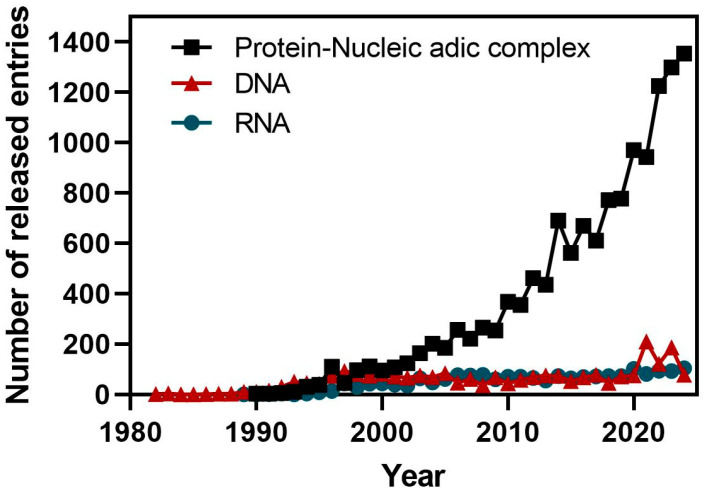
Time course of the deposition statistics in the PDB database showing the number of released entries by year for protein–nucleic acid complexes, and for DNA and RNA molecules (source: PDB statistics).

The availability of ncRNA structures in public domain databases such as the PDB [63], is very limited when compared to proteins [64]. The reasons for this lack of experimental information are complex to analyze, but they should rely on the nature of RNA molecules that limit their analysis by wet-lab protocols. RNA molecules are highly flexible and adopt diverse secondary and tertiary structures. Moreover, RNA often exists in multiple stable conformations, complicating efforts to determine a single, well-defined structure. An additional factor is related to the historical emphasis placed on studying protein structures, as they are directly linked to specific biological events such as enzymatic catalysis [65]. This has also led to a disproportionate number of resources allocated to protein structural studies compared to RNA. Interestingly, an exponential growth of available structures from protein–nucleic acid complexes has been experienced in the last decade due to technical improvements in structure determination by X-ray crystallography and cryo-electron microscopy (Figure 3). Among these structures, there are several entries that include fragments or complete ncRNAs in complex with proteins.

**Figure 4:**
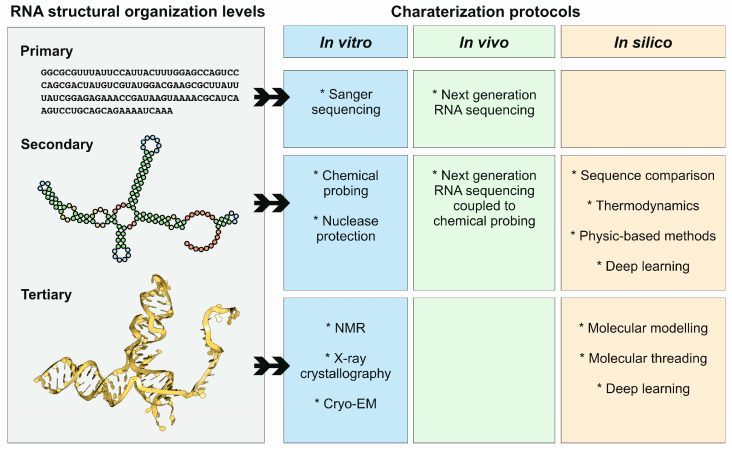
Structural organization levels of RNA molecules, and protocols used for their characterization, in vitro, in vivo, and in silico. Note that the different protocols and methods are highly connected, and in many cases are used together as synergistic approaches.

The structure of ncRNAs and other functionally relevant RNAs can be studied by different approaches if a characterization of the secondary or tertiary structures is required. Compared to proteins, RNA molecules can be structurally characterized by a more diverse group of methods due to their chemical nature (Figure 4). In fact, classical methods such as nuclear magnetic resonance, X-ray crystallography, and cryo-electron microscopy can be used for in vitro characterization of RNA tertiary structure. Additionally, the secondary structure of RNA molecules can be characterized in vivo and, in a genome-wide manner, by combining chemical probing and next-generation sequencing. Moreover, computer-based methods can also be applied for the prediction and modeling of RNA secondary and tertiary structures.

### 4.1. X-Ray Crystallography

X-ray crystallography is a classical technique in structural biology, enabling the elucidation of three-dimensional structures at atomic resolution through X-ray diffraction [66]. The obtention of meaningful X-ray diffraction patterns requires the use of molecular crystals, ordered 3D lattices composed of the spatial repetition of molecular units following geometrical rules [67]. Despite its importance, RNA crystallization faced important challenges due to the intrinsic properties of RNA molecules, such as their structural flexibility, heterogeneity, and biochemical instability [68]. RNA molecules are highly dynamic and flexible, often adopting multiple conformations due to their inherent base-pairing and stacking interactions. This conformational heterogeneity often prevents the formation of ordered crystalline lattices. The presence of non-canonical interactions and tertiary motifs can further exacerbate this challenge by creating structurally diverse populations of RNA. RNA is also prone to degradation by RNAses and spontaneous hydrolysis under physiological conditions [69]. The susceptibility of RNA to degradation complicates its preparation and handling during crystallization experiments [66]. Another important factor that could prevent RNA crystallization is the relative molecular size. Whereas short RNA molecules (<20 nucleotides) may not contain sufficient structural complexity to support crystal lattice formation, long RNA sequences, which are very frequent in ncRNAs (>100 nucleotides), often exhibit excessive flexibility and heterogeneity. Additionally, the sequence composition, such as high uridine or cytidine content, can influence RNA’s propensity to crystallize [70]. RNA tends to form poorly ordered crystals due to suboptimal packing interactions between molecules. The dominance of electrostatic repulsion between negatively charged phosphate backbones may prevent the close packing required for high-resolution X-ray diffraction [67]. Even when high-quality RNA crystals are obtained, determining phases for X-ray diffraction can be challenging due to the lack of established protocols for introducing heavy-atom derivatives into RNA crystals, which are necessary for phase retrieval using isomorphous replacement (MIR) or multi-wavelength anomalous dispersion (MAD) methods [71].

Some strategies have been developed to increase the probability of successful crystallization of RNA molecules, which have been applied to ncRNAs. The main strategy used was to minimize the RNA conformational heterogeneity by rational design of RNA constructs that can enhance crystallization [69]. These include the truncation of non-essential regions, introducing stabilizing mutations (e.g., replacing uridines with pseudouridines), or incorporating locked nucleic acids (LNAs) that can reduce flexibility and conformational heterogeneity. Moreover, some tertiary structure scaffolds can be engineered, such as kissing loops or tetraloops, to promote the formation of uniform RNA structures that could facilitate crystallization. Incorporation of 2′-O-methyl or phosphorothioate groups can also enhance RNA stability by reducing susceptibility to hydrolysis and RNase degradation [72]. Adding divalent cations (e.g., Mg^2^⁺, Mn^2^⁺) or polyamines can mitigate electrostatic repulsion between phosphate groups, facilitating tighter packing in crystals. In any case, it is evident that RNA often crystallizes more readily as part of an RNA–protein complex than as a standalone molecule [70]. Proteins can serve as scaffolds that stabilize RNA tertiary structures and promote crystal lattice formation. Co-crystallization with RNA-binding proteins or using engineered proteins with high affinity for RNA motifs is a commonly employed strategy for structural studies of ncRNAs.

X-ray crystallography was used for solving the structure of small and compact ncRNAs such as catalytic prokaryotic ncRNAs. Ribozymes are a classic example of ncRNAs whose structures have been resolved using X-ray crystallography. The hammerhead ribozyme, which catalyzes site-specific RNA cleavage, was among the first ribozymes to have its structure determined [73]. Early studies used X-ray crystallography to reveal the catalytic core’s architecture, including the specific interactions between nucleotide residues critical for its function [74]. These findings provided a molecular basis for understanding RNA catalysis and paved the way for engineering RNA-based catalysts. Another example is riboswitches, regulatory elements in messenger RNAs that control gene expression by binding to small metabolites. X-ray crystallography has been instrumental in uncovering the binding pockets and conformational changes of various riboswitches [75]. For instance, the structure of the thiamine pyrophosphate (TPP) riboswitch was determined at high resolution, revealing how the RNA specifically recognizes the TPP molecule through hydrogen bonding and van der Waals interactions [76]. These studies elucidated the structural basis for ligand-induced RNA folding and regulation.

Small and medium-sized ncRNAs from eukaryotic organisms have also been crystallized, and their structures solved by X-ray diffraction. Pioneer examples include the resolution of the structure of tRNAs from yeast and humans [77]. More recently, X-ray diffraction was used to characterize the structure of specific processing products generated from lncRNAs. The primary transcripts of some lncRNAs, such as MALAT1 and MENβ, contain a tRNA-like structure at their 3′ end, which are processed and cleaved by tRNA processing enzymes RNase P and RNase Z. This maturation pathway generates a cytoplasmic tRNA-like small RNA, known as mascRNA for MALAT1 and menRNA for MENβ, along with mature nuclear retained MALAT1 and MENβ lncRNAs [78]. The molecular structures of menRNA and mascRNA have been solved by X-ray crystallography [79].

### 4.2. Cryo-Electron Microscopy

**Figure 5:**
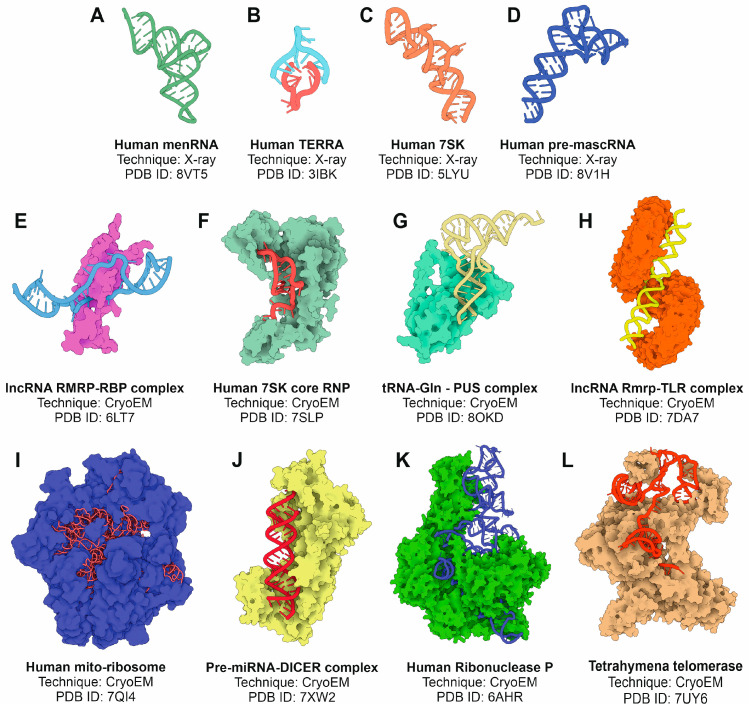
Selected examples of experimentally determined tertiary structures of ncRNAs or complexes of ncRNAs with proteins using X-ray crystallography and cryo-EM methods. (**A**) Human memRNA; (**B**) human telomere associated RNA; (**C**) human 7SK RNA; (**D**) human pre-mascRNA; (**E**) complex between lncRNA Rmrp and an RNA-binding protein; (**F**) human 7SK core ribonucleoprotein; (**G**) glutamine-tRNA in complex with PUS protein; (**H**) lncRNA Rmrp in complex with TLR; (**I**) human spliceosome; (**J**) human pre-miRNA complex with Dicer endonuclease; (**K**) human ribonuclease P complex; and (**L**) Tetrahymena telomerase. All the figures were prepared from the atomic coordinate data extracted from the PDB database and the 3D Protein Imaging application [100].

Cryo-electron microscopy (cryo-EM) has emerged as a transformative technique in structural biology, offering unique advantages for studying RNA molecules, including ncRNAs [80]. Cryo-EM’s ability to resolve structures in a near-native environment, without requiring crystallization, has significantly advanced our understanding of ncRNAs, even those with challenging biochemical properties. Recent technological developments in cryo-EM, such as direct electron detectors, advanced computational algorithms, and phase plate technology, have greatly improved resolution and contrast, enabling near-atomic structural determination of large and complex ncRNAs as well as ncRNA-protein assemblies [81]. Cryo-EM circumvents the size and flexibility constraints often encountered with other techniques such as X-ray crystallography. Cryo-EM has been successfully applied to elucidate the structures of several ncRNAs and their complexes, offering detailed insights into their mechanisms of action [82] (Figure 5).

However, when applied to RNA molecules, cryo-EM also faces some limitations that could hinder its accuracy and applicability. RNA molecules exhibit inherently low contrast in cryo-EM images due to their lower electron scattering compared to proteins. This makes it difficult to resolve RNA structures, particularly in small or isolated RNA molecules. Additionally, RNA is highly susceptible to radiation damage, which can lead to structural degradation before sufficient data can be collected. RNA molecules often adopt multiple conformations due to their inherent flexibility [83]. Unlike proteins, which typically fold into stable 3D structures, RNA can exist in a dynamic ensemble of states. This structural heterogeneity complicates image classification and 3D reconstruction. For instance, cryo-EM studies on riboswitches have revealed multiple conformational states, making it challenging to assign a single definitive structure [84]. Single-particle cryo-EM relies on the computational alignment of thousands to millions of individual particles to reconstruct a high-resolution structure. Due to their elongated and often asymmetric shapes, RNA molecules pose difficulties in alignment, leading to lower resolution reconstructions. This problem is particularly evident in studies of small non-coding RNAs, where obtaining well-aligned particles is challenging. Even at near-atomic resolution, cryo-EM faces challenges in resolving fine RNA features such as base stacking, hydrogen bonding, and minor groove interactions. These interactions are crucial for RNA folding and function but can be difficult to interpret due to limited resolution. In consequence, it would be desirable to combine cryo-EM with complementary techniques, such as chemical probing or molecular dynamics simulations, to provide more comprehensive insights into ncRNA structure and function [81].

One of the landmark applications of cryo-EM to elucidate the function of ncRNAs was the resolution of the spliceosome structure. The spliceosome is a dynamic ribonucleoprotein complex responsible for the precise removal of introns from pre-mRNA transcripts. High-resolution cryo-EM studies on the yeast spliceosome by Yan and colleagues in 2015 and 2016 have provided detailed insights into its structural organization during various stages of the splicing cycle [85,86]. Cryo-EM analysis solved the structure of the catalytic center of the spliceosome, formed by intricate RNA–RNA and RNA–protein interactions. Key elements include the U2/U6 snRNA duplex, which forms a three-helix junction critical for catalysis, and the U5 snRNA, which interacts with the 5’ and 3’ splice sites to align the exons for ligation [85]. Additional details of the dynamics of the yeast spliceosome were also determined by cryo-EM experiments, showing that the spliceosome undergoes significant conformational rearrangements to facilitate the two-step splicing reaction where ncRNAs have an important role. In the transition from the Bact (activated) to the C* (catalytically activated) complex, the first and second transesterification reactions are possible due to structural rearrangements that orient the pre-mRNA substrate and the catalytic components of the spliceosome. These rearrangements are mediated by ATP-dependent RNA helicases, which remodel RNA–RNA and RNA–protein interactions to drive the splicing cycle forward [87]. Bertram and coworkers reported a 3D cryo-EM structure of the human pre-catalytic spliceosome B complex. This structure revealed that the U2 snRNP-containing head domain is connected to the main body of the B complex via three primary bridges [88,89]. In a separate study, Zhang and coworkers presented the cryo-EM structure of the human spliceosome just before exon ligation, known as the C* complex. This structure provided insights into the arrangement of splicing factors surrounding the catalytic core of the complex [90]. These studies highlight both conserved and unique features of the human spliceosome compared to its yeast counterpart. For instance, while the overall architecture of the catalytic U2-U6 ribonucleoprotein core is similar between species, the human spliceosome exhibits specific structural adaptations, such as the involvement of metazoan-specific proteins that stabilize key protein and RNA regions.

Telomerase is a ribonucleoprotein complex that extends telomeres, the protective ends of linear chromosomes, by adding repetitive nucleotide sequences. Cryo-EM studies have elucidated the structural characteristics of telomerase in both humans and the ciliate *Tetrahymena thermophila*, providing insights into their functional mechanisms. In 2018, Jiang and colleagues reported the cryo-EM structure of the *Tetrahymena* telomerase holoenzyme at a resolution of approximately 4.8 Å [91]. This study revealed that the catalytic core of the enzyme consists of TERT and telomerase ncRNA (TER). The ncRNA component adopts a conserved pseudoknot structure, essential for its function. In 2021, researchers achieved a significant milestone by constructing the first atomic model of the human telomerase complex using cryo-EM, reaching a resolution of approximately 3.0–4.0 Å [92]. This structure revealed a bilobular architecture comprising a catalytic core lobe, containing the telomerase reverse transcriptase (TERT) and the telomerase ncRNA (hTR), and an H/ACA ribonucleoprotein (RNP) lobe, formed by two sets of heterotetrameric H/ACA proteins and the Cajal body protein TCAB1 [92]. While both human and *Tetrahymena* telomerases share a conserved catalytic core architecture surrounding the ncRNA, differences exist in their accessory components and regulatory mechanisms. The specific molecular interactions involved in telomerase recruitment and regulation differ between the two species, reflecting evolutionary divergence [92,93].

Recent advancements in cryo-EM have also provided detailed insights into the biogenesis and mechanism of action of small regulatory RNAs such as miRNAs, based on the structural characterization of RNA–protein complexes [94]. Drosha, an RNase III enzyme, initiates miRNA maturation by cleaving primary miRNAs (pri-miRNAs) into pre-miRNAs within the nucleus. It functions as part of the microprocessor complex, which includes the DGCR8 protein. High-resolution cryo-EM structures of the full Drosha-DGCR8 complex bound to pri-miRNA have provided insights into its functional domains and interactions. The DGCR8 component contains an RNA-binding domain that recognizes and binds pri-miRNAs, positioning them for precise cleavage by Drosha [95]. This interaction is crucial for the accurate processing of pri-miRNAs into pre-miRNAs, a critical step in miRNA biogenesis. Dicer, another RNase III enzyme, is responsible for processing precursor miRNAs (pre-miRNAs) into mature miRNAs at the cytoplasm. In 2018, Liu and coworkers solved the structure of human Dicer in complex with a pre-miRNA substrate by cryo-EM [96]. Additional studies revealed that human Dicer, in association with the trans-activation response RNA-binding protein (TRBP), forms a complex that interacts with pre-miRNA [97,98]. The structure highlighted two distinct conformations of the pre-miRNA within the complex: one adopting a perfect base-paired A-form helix and the other exhibiting a partially splayed structure. These conformations suggest a mechanism by which Dicer accommodates various pre-miRNA structures to ensure precise cleavage. Moreover, the DExD/H-box helicase domain of Dicer was observed to have a C-shaped architecture, implicating its role in regulating Dicer’s activity during miRNA processing [96,97]. Cryo-EM has also been used to characterize other proteins involved in the miRNA biogenesis pathway, such as the Exportin 5, a protein required for the proper transport of pre-miRNAs from the nucleus to the cytoplasm. The structure of the complex between Exportin 5 and a pre-miRNA has been solved by Cryo-EM and has been essential to understand the transport mechanism and the unspecific accommodation of miRNA precursors into the core of the shuttle protein [99].

### 4.3. In Vivo Methods

RNA secondary structures play crucial roles in the function and regulation of non-coding RNAs (ncRNAs). The in vivo determination of RNA secondary structures is particularly important because cellular environments introduce unique influences, such as molecular crowding, protein binding, and chemical modifications, which can differ significantly from in vitro conditions. Chemical probing methods have emerged as indispensable tools for characterizing RNA secondary structures in their native cellular contexts. These approaches exploit the reactivity of RNA nucleotides with small molecules, which differ depending on the nucleotide’s structural context (single-stranded, paired, or tertiary interactions). In vivo probing captures RNA folding in the presence of cellular cofactors, metabolites, and interacting proteins, providing a more accurate reflection of functional RNA structures. Coupling probing with next-generation sequencing enables transcriptome-wide analysis of RNA structures. The dynamic structural states of ncRNAs can be monitored, shedding light on their roles in processes like translation regulation or splicing [101].

Chemical probing methods have been extensively used for the determination of RNA structures, both in vitro and in vivo. However, several intrinsic limitations hinder their application in accurately resolving RNA conformations within the complex cellular environment. These limitations stem from factors related to the chemical reactivity of the probes, the dynamic nature of RNA structures, cellular conditions, and data interpretation challenges. Many RNA probing reagents, such as dimethyl sulfate (DMS), 1-methyl-7-nitroisatoic anhydride (1M7), and kethoxal, react specifically with accessible nucleotides in structured RNA. However, their specificity is often influenced by factors unrelated to RNA structure, such as local chemical environment, steric hindrance from proteins, and the presence of molecular crowding in vivo. Additionally, some reagents may have off-target effects or induce structural perturbations, thereby introducing artifacts in the probing data. RNA structures are highly dynamic, adopting multiple conformations depending on cellular conditions, protein interactions, and ligand binding. Chemical probing methods provide only a snapshot of RNA structure at a given time point, failing to capture transient or low-abundance conformations. Moreover, ensemble-averaged chemical probing data may obscure coexisting structural states, leading to inaccurate structural interpretations. In vivo RNA probing is influenced by cellular factors such as ion concentrations, macromolecular crowding, and metabolic activities, which differ significantly from in vitro conditions [102]. Additionally, the presence of RNA-binding proteins (RBPs) can alter the accessibility of nucleotides, complicating the interpretation of reactivity data as a direct readout of RNA secondary structure. Chemical probing experiments generate large datasets that require extensive computational analysis for structure modeling. Signal deconvolution, background correction, and normalization methods introduce additional sources of error, particularly when comparing in vitro and in vivo datasets. Furthermore, the interpretation of reactivity data relies on computational algorithms that may oversimplify RNA folding rules, leading to discrepancies between predicted and actual structures [103].

**Figure 6:**
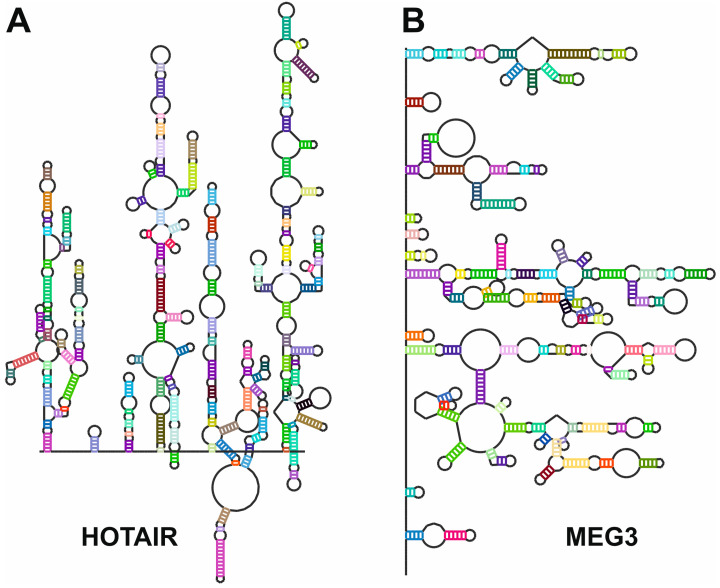
Secondary structures of (**A**), HOTAIR [108] and (**B**), MEG3 [56] lncRNAs as determined by chemical probing followed by next-generation sequencing. Sequences and secondary structures were retrieved from the lncRNA-folding repository [109]. Secondary structure maps were generated by the RNArtist software v.1.0 (https://github.com/fjossinet/RNArtist, accessed on 31 January 2025).

The original chemical probing method, SHAPE-seq, involves the use of chemical reagents, such as 1-Methyl-7-nitroisatoic anhydride (1M7) or N-methylisatoic anhydride (NMIA), which preferentially react with flexible nucleotides in single-stranded regions of RNA. These reagents modify the 2′-hydroxyl group of the ribose ring, creating adducts that interfere with reverse transcription. By analyzing reverse transcription termination or mutation events through next-generation sequencing, the structural reactivity of RNA can be quantified. This method is well-suited for probing RNA in vitro but is limited to addressing in vivo dynamics and structural heterogeneity. Variants such as icSHAPE (in vivo click SHAPE) were developed to overcome the limitations of traditional SHAPE-seq in analyzing RNA structure in live cells [104]. This method employs a cell-permeable SHAPE reagent, such as NAI-N3, which can penetrate cellular membranes to modify RNA in its native cellular context. After chemical modification, the azido moiety in NAI-N3 enables “click chemistry” to biotinylate modified RNA, allowing selective enrichment of labeled molecules. icSHAPE is instrumental in studying RNA structures influenced by protein interactions, cellular compartmentalization, and environmental stimuli. Another recent modification of chemical probing methods named SHAPE-MaP (mutational profiling) is a SHAPE-seq variant that directly integrates chemical modification information into cDNA during reverse transcription [105]. Instead of terminating at modification sites, the reverse transcriptase incorporates mutations or misincorporations at modified nucleotides. These mutations are subsequently identified via sequencing [106]. SHAPE-MaP has several advantages, including reduced noise, improved quantitative accuracy, and compatibility with both in vitro and in vivo conditions. It also facilitates probing of RNA structure under a wide range of conditions, including complex environments like cell lysates [107]. Chemical probing techniques coupled with RNA sequencing have been applied for the secondary structure characterization of selected lncRNAs (Figure 6).

Initial studies were focused on the structural characterization of relatively small lncRNAs, such as the steroid receptor activator RNA (SRA), with approximately 800 nt in length. SRA is a co-activator of several sex hormone receptors, being strongly associated with some tumors. SHAPE-seq and RNAse probing were used to characterize the evolutionarily conserved structural domains of SRA [110]. In the following years, the development of new variants of the chemical probing techniques together with the improvement of the analysis software allowed the structural characterization of more complex lncRNAs. For instance, the structure of MALAT1 (approximately 8400 nt in length), a long non-coding RNA (lncRNA) implicated in gene expression regulation, was probed using SHAPE-seq to identify its structural domains and interactions, which are critical for its localization and function. In a recent work, the application of SHAPE-MaP for the characterization of MALAT1 in green monkeys and humans revealed a conserved structural pattern between the lncRNA in both species, despite the sequence differences [111]. Another interesting example is NEAT1, an lncRNA essential for the formation and maintenance of paraspeckles, nuclear bodies involved in RNA metabolism and processing [112]. SHAPE-seq and DMS probing were applied to NEAT1, revealing its highly modular structure and the existence of specific regions that were characterized as essential for paraspeckle assembly [113]. This study also contributed to describing the evolutionary divergences present in the structures of NEAT1 between humans and mice and proposed a functional model of NEAT1 lncRNA where the structural elements that contribute to paraspeckle assembly are based on long-range interactions rather than in conserved domains [113]. SHAPE-seq was also used for the structural characterization of HOTAIR (HOX transcript antisense RNA), an lncRNA involved in chromatin remodeling and gene silencing, particularly by guiding the polycomb repressive complex 2 (PRC2) to specific genomic loci [108]. Interestingly, HOTAIR structure reveals a complex degree of structure organization that is comparable to well-folded ncRNAs such as the group II intron or ribosomal RNAs. HOTAIR comprises four independent folding units, where two of them are predicted as protein-binding domains [108]. A similar modular organization was also observed in MEG3 (maternally expressed gene 3), a lncRNA that acts as a tumor suppressor by modulating the p53-mediated response. In this lncRNA, the folding is stabilized by distal motifs that interact by forming pseudoknot structures. A detailed SHAPE-seq study by Uroda and coworkers showed that mutations able to disrupt these pseudoknot structures impaired the MEG3-dependent stimulation of the p53 pathway by decreasing MEG3 stability [56].

Other documented examples of the application of chemical probing technologies include the structural characterization of growth arrest specific 5 lncRNA (GAS5) by SHAPE-MaP [114], the application of SHAPE-seq and DMS-seq for the dissection of the secondary structure elements of the Xist lncRNA [115], and the studies of the dynamics of telomeric repeat-containing RNAs as important factors contributing to chromosome stability [93].

### 4.4. In Silico Methods

The hierarchical nature of RNA folding, where secondary structure elements are formed prior to their combination into higher-order tridimensional structures, makes secondary structure prediction very relevant for understanding RNA function [116]. Many of the in silico approaches rely on the nearest-neighbor thermodynamic model to predict secondary structures by minimizing free energy. However, the accuracy of these models is limited by the partial availability of thermodynamic parameters for RNA molecules, especially for non-canonical base pairs and tertiary interactions, and also by the assumption that RNA adopts the minimum free energy (MFE) structure, which is not always true in vivo due to kinetic folding pathways and environmental factors [29]. While this assumption often holds, it overlooks kinetic folding pathways and metastable states, which can be functionally relevant. Consequently, predictions may not always capture the biologically active structure [21]. Moreover, our understanding of RNA folding rules is incomplete, particularly for complex motifs such as pseudoknots, which involve interactions between non-adjacent regions of the RNA molecule. Although some algorithms attempt to account for pseudoknots, these structures remain challenging to predict accurately due to their computational complexity and lack of comprehensive thermodynamic parameters [117]. RNA folding is also influenced by the cellular environment, including ionic conditions, molecular crowding, and interactions with proteins or other RNAs. Computational tools often operate under simplified conditions that do not fully replicate the cellular milieu, leading to differences between predicted and actual structures [27,28]. For instance, most secondary structure prediction tools assume “standard” conditions, such as neutral pH and absence of molecular crowding. In a biological system, the cellular environment contains proteins, ions, and metabolites that significantly influence RNA folding. Computational methods struggle to incorporate these dynamic effects, leading to discrepancies between in silico predictions and experimental observations [109].

#### 4.4.1. Methods for RNA Secondary Structure Prediction

**Figure 7:**
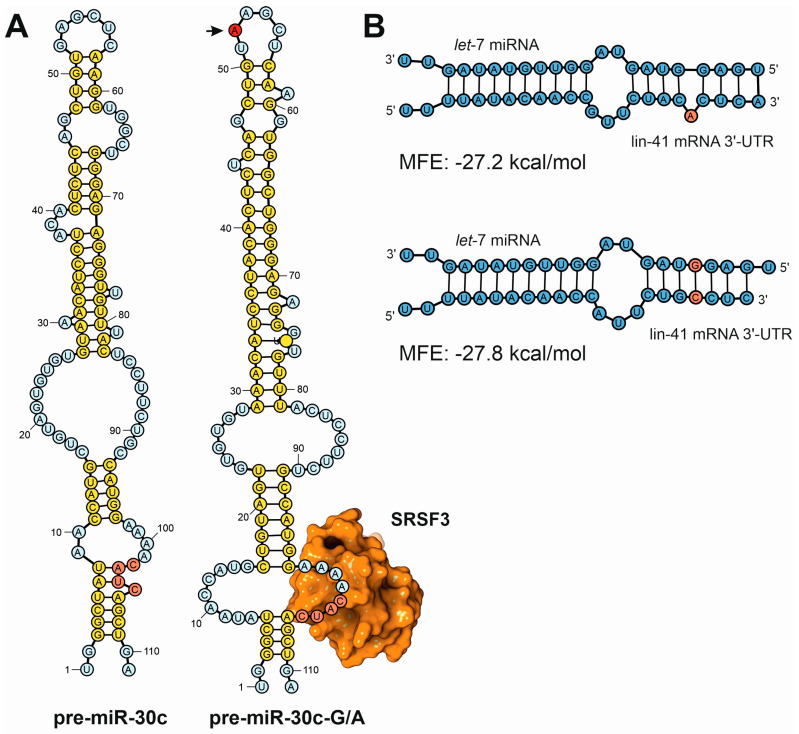
Examples of secondary structure predictions applied to the study of the influence of sequence variants in the functions of ncRNAs. (**A**) Fernández and coworkers used a combination of in silico prediction techniques with experimental validation to demonstrate the differences in the processing efficiency of miRNA precursors induced by single point mutations [123]. This depiction shows the computer predictions of the secondary structure of the human pre-miR-30c canonical precursor (pre-miR-30c) compared to the A52G mutant (pre-miR-30c-G/A, mutation position depicted by an arrow). In the mutated precursor, the base of the loop presents an open structure that exposes the CNNC canonical sequence for SRSF3 binding, ensuring a more efficient processing by the nuclear DGCR8-Drosha complex. (**B**) Detailed analysis of the computer predictions of miRNA–mRNA hybrids established between let-7 miRNA and lin-41 3′-UTR, showing the differences in the estimated Gibbs function in two miRNA targets according to the presence of sequence bulges generated by sequence variants [124].

RNA secondary structure elements can be predicted by diverse families of algorithms that are designed with different strategies [118]. Thermodynamic methods predict RNA secondary structure by minimizing the free energy of folding by applying thermodynamics and base-pairing probabilities. Representative computer tools that use thermodynamics for RNA secondary structure prediction are RNAfold [119], RNAstructure [120], Sfold [121], and Mfold [122]. These methods are especially efficient for the prediction of secondary structures in small RNAs (less than 500 nt). Thermodynamic-based methods have been extensively employed for the characterization of secondary structures in miRNA precursors. For instance, Fernández and coworkers showed how the genetic variants of the miR-30c precursor modify the secondary structure of the processed hairpin loop, altering the processing efficiency of the Drosha-DGCR8 complex and the levels of mature miRNA (Figure 7A). The computer prediction of secondary structures of the different precursors was correlated to the processing efficiency by the Drosha-DGCR8 complex of the different hairpin loops, showing a clear correlation between structural variants, RNA stability, and processing outcome [123]. Moreover, the same prediction methods were used to study the influence of the secondary structure elements present in the 3’-UTR of mRNA transcripts and their relationship to miRNA regulatory activity. Analyzing different mutants of the 3’-UTR of the lin41 gene from *Caenorhabditis elegans*, Long and coworkers predicted the secondary structure of the let-7 target hybrids using the Sfold tool and validated the regulatory effect of the miRNA (Figure 7B). The work determined a potent effect on target recognition driven by the presence or absence of secondary structure elements in the miRNA target [124]. Additionally, comparative sequence analysis methods can also be used to determine secondary structures of ncRNAs. In this approach, covariation analysis can identify the paired residues that evolve in a coordinated manner, suggesting base-pairing interactions [125]. Databases such as Rfam were built based on comparative sequence analysis to annotate ncRNA families and predict conserved secondary structure elements in ncRNAs [126].

Recently, machine learning techniques have transformed RNA secondary structure prediction by leveraging large datasets and powerful algorithms to identify complex patterns. Machine learning models, particularly deep learning approaches, can integrate diverse sources of data, including primary RNA sequences, chemical probing experiments, and evolutionary conservation [127]. These models bypass some of the limitations of thermodynamic methods by learning directly from empirical data. However, the application of these methods to the prediction of secondary structures of ncRNAs is not widespread [128]. Among the different deep learning methods applied to the prediction of RNA secondary structures, supervised models are trained on datasets of RNA sequences with experimentally validated secondary structures. Convolutional neural networks (CNNs) and recurrent neural networks (RNNs) have been employed to capture local and global dependencies in RNA sequences. For example, the SPOT-RNA model uses a combination of CNNs and long short-term memory (LSTM) networks to predict base-pairing probabilities with high accuracy [129]. In graph neural network models (GNNs), RNA molecules can be represented as graphs, with nucleotides as nodes and base-pairing or spatial proximities as edges. GNNs are particularly suited for modeling such relationships. RNAformer, for example, uses graph attention mechanisms to predict secondary structure while accounting for long-range interactions [130].

#### 4.4.2. Methods for RNA Tertiary Structure Prediction

RNA tertiary structure prediction remains a formidable challenge in computational biology due to several intrinsic and extrinsic factors. These difficulties stem from the complex nature of RNA molecules, the limitations of current computational methodologies, and the intricate interplay of physicochemical forces governing RNA folding [131]. Intrinsic challenges related to RNA structure include its structural flexibility, extensive presence of long-range interactions and multitude of stable conformations [132]. Computer methodologies and protocols for RNA tertiary structure prediction are limited mainly by the suboptimal accuracy of current force fields tailored for RNA, which prevents a proper energy-based modeling of RNA structures [133]. Moreover, the large size of specific families of ncRNA molecules, such as lncRNAs, and their intricate interactions require extensive computational resources, increasing the time required for accurate calculations. The scientific community, aware of all these experimental hurdles, has established some projects like the RNA-Puzzles initiative, started in 2010, that emerged as a collaborative effort to advance the understanding of RNA structure prediction. The project was inspired by the success of similar initiatives like CASP (Critical Assessment of Protein Structure Prediction) in the field of protein modeling [134]. RNA-Puzzles serves as a blind benchmarking platform where participants are tasked with predicting RNA structures based solely on sequence information, without prior knowledge of experimentally determined structures. These predictions are then evaluated against experimentally derived models, enabling the comparison of different computational approaches [135].

Among the wide range of prediction methods used to model RNA 3D structure, homology modeling relies on the use of structural similarity between RNAs to predict their 3D structure. Tools like ModeRNA are based on the use of already known RNA structures as templates to predict the tertiary conformation of homologous sequences [136]. Other methods, such the fragment assembly algorithms, predict RNA tertiary structures by assembling smaller structural motifs, combining them into a higher-order structure. Pioneer algorithms such as the MC-Fold/MC-Sym pipeline are good examples that were used to predict the 3D structures of different pre-miRNAs [137]. More recently, the same experimental strategy was used to develop algorithms such as FARFAR2, a new fragment assembly of RNA with full-atom refinement [138]. This method was successfully applied for the structural prediction of small and well-structured ncRNAs such as ribozymes and riboswitches [139].

Achieving high accuracy in RNA structure prediction often necessitates combining information from different structural scales. Multiscale and hybrid methods that integrate secondary and tertiary structure prediction techniques have emerged as powerful approaches to address this challenge. RNAComposer is a prominent example that combines thermodynamic principles with fragment assembly techniques to predict RNA 3D structures. RNAComposer uses secondary structure predictions as a starting point, ensuring that the base-pairing interactions are thermodynamically feasible [140]. After these predictions, the software maps predicted secondary structures onto a database of known RNA motifs to assemble a final 3D model. The hybrid methodology allows RNAComposer to incorporate both local base-pairing information and global structural motifs, yielding accurate 3D models [141]. Hybrid and multiscale methods have demonstrated significant improvements in prediction accuracy. They enable the modeling of complex RNA structures, including ribozymes, riboswitches, and large non-coding RNAs [142]. The integration of secondary and tertiary prediction methods is particularly valuable for modeling large RNA molecules where accurate tertiary structure prediction is computationally challenging [143].

Molecular dynamics is a computational technique that models the physical movements of atoms and molecules over time. It relies on solving Newton’s equations of motion for a system of particles, where the interactions are described by a potential energy function, or force field. For RNA, MD simulations provide insights into folding pathways, conformational flexibility, and the effects of sequence mutations on structure and function [144]. Accurate modeling of RNA requires specialized force fields that capture the unique chemical and physical properties of nucleotides to accurately reproduce RNA’s backbone conformations, hydrogen bonding patterns, and base-stacking interactions [145,146]. RNA folding involves a hierarchical process, where local secondary structures such as helices and loops form first, followed by the establishment of tertiary contacts. MD simulations can capture these folding events by simulating the molecule in explicit solvent conditions, mimicking the cellular environment. Enhanced sampling techniques, such as replica exchange molecular dynamics (REMD) and meta-dynamics, are often employed to overcome the high energy barriers associated with RNA folding and sample a broader conformational space [147]. While all-atom MD simulations offer high-resolution insights, their computational cost limits their implementation in long time scales required to study phenomena such as RNA folding [148]. Coarse-grained models, which simplify RNA representation by grouping atoms into larger units, enable simulations of larger systems and longer timescales [149]. Due to the specific transient nature of the miRNA-based regulation, molecular dynamics has been employed for the characterization of the stability of miRNA–mRNA complexes [150]. Moreover, molecular dynamics simulations were also used to study the stability of the heterologous complex established between the miRNAs, their targets, and the core of the RNA-induced silencing complex (RISC), Ago2 [151]. Interestingly, all the theoretical simulations performed during miRNA–mRNA interactions concluded that the spectrum of a miRNA’s potential targets is different from what is currently anticipated by the canonical miRNA seed model. These findings could explain the relative promiscuity of miRNA regulatory action and its transient nature, which could be based on the observed flexible regulatory effect over mRNA transcripts [150,151].

## 5. Perspectives and Further Developments

The application of structural biology techniques to non-coding RNAs (ncRNAs) represents a rapidly advancing frontier, driven by the recognition of ncRNAs’ diverse biological roles. The development of advanced structural biology and new devices and protocols related to cryo-electron microscopy (cryo-EM) and X-ray crystallography methods has enabled high-resolution characterization of complex ncRNA structures. These techniques have revealed intricate folding patterns and tertiary interactions critical to ncRNA function. More recently, integrative approaches combining structural data with computational modeling and single-molecule experiments have expanded our capacity to resolve dynamic and transient ncRNA conformations. Cryo-EM has revolutionized the field by allowing the study of large ribonucleoprotein (RNP) complexes, such as the spliceosome and ribosome, at near-atomic resolution. Simultaneously, advancements in computational RNA structure prediction tools, powered by machine learning, have enhanced the accuracy of secondary and tertiary structure predictions. These tools complement experimental techniques, enabling the characterization of challenging ncRNAs and their complexes. Looking ahead, several key areas promise to drive further advancements in this field.

- Integration of multiscale data: combining atomic-level structural information with systemic data on ncRNA localization, interaction networks, and dynamics will enable a holistic understanding of ncRNA function. Hybrid approaches integrating cryo-EM and chemical probing with single-cell RNA sequencing and spatial transcriptomics are particularly promising.
- High-throughput structural characterization: automation in cryo-EM and microfluidics-based methods for RNA crystallography could facilitate the high-throughput determination of ncRNA structures, accelerating the discovery of novel functional motifs.
- Dynamic and contextual studies: capturing ncRNA structures in their native cellular environment remains a significant challenge. Emerging techniques, such as cryo-electron tomography (cryo-ET) and in situ structural studies, aim to bridge this gap by visualizing ncRNAs within intact cells.
- Functional modulation and rational drug design: structural insights into ncRNAs have profound implications for drug discovery. Small molecules targeting ncRNA structures or their interactions with proteins could provide new therapeutic avenues for diseases associated with dysregulated ncRNAs, such as cancer and neurodegenerative disorders.
- Evolutionary perspectives: structural comparisons across species can reveal conserved motifs and inform functional hypotheses. Integrating structural biology with evolutionary genomics will help identify universally important ncRNA structures and their roles in diverse organisms.
- Artificial intelligence: the use of deep-learning approaches to infer ncRNA structure and function will increase our understanding of their functions, increasing the knowledge about the molecular players related to cell physiology and human disease.
